# Presumptive Zoonotic Kerion by *Nannizzia gypsea*: Case Report

**DOI:** 10.3389/fvets.2021.718766

**Published:** 2021-08-24

**Authors:** Deborah Cruciani, Manuela Papini, Sayra Broccatelli, Francesco Agnetti, Sara Spina, Ylenia Natalini, Silvia Crotti

**Affiliations:** ^1^Centro Specialistico Patologie Micotiche, Istituto Zooprofilattico Sperimentale dell'Umbria e delle Marche “Togo Rosati”, Perugia, Italy; ^2^Clinica Dermatologica di Terni, Università degli Studi di Perugia, Perugia, Italy; ^3^Igiene degli Allevamenti e delle Produzioni Zootecniche, Dipartimento di Prevenzione, Unità Sanitaria Locale Umbria 2, Terni, Italy

**Keywords:** kerion, *Nannizzia gypsea*, child, dog, case report

## Abstract

*Nannizzia gypsea* (formerly *Microsporum gypseum*) belongs to geophilic dermatophytes, fungi involved in keratin degradation in the soil; however, they are also found in dogs and cats. Transmission to humans can occur directly by contact with soil, but indirect transmission *via* domestic animals is reported too. The exact source of the infection is usually difficult to assess and in most cases only hypothesised and rarely investigated. This case report describes a kerion caused by *N. gypsea* in a 2-year-old boy, where the contagion was probably secondary to domestic healthy carrier dogs. A “One-Health” approach involving human dermatologists and veterinarians, combined with the use of conventional and molecular-based techniques, allowed tracing of the epidemiological chain and managing of not only the treatment but also the prevention of a recurrence. The child's lesion began to regress after about 8 weeks of treatment with both systemic and topical therapy, while the dogs were given chlorhexidine and miconazole baths. No recurrences nor new infections occurred, demonstrating the effectiveness of the strategies used.

## Introduction

Dermatophytes are filamentous fungi that invade keratinized tissues (i.e. skin, hair and/or nails) of humans and other animals, causing infections known as dermatophytosis (1). Dermatophytes play an important role in animal and human health due to their zoonotic potential (2). According to their natural habitat, dermatophytes are usually categorised into anthropophilic, zoophilic and geophilic species (1) even if these ecological groups are not sharply separated (3). Nowadays, pets bring social and health-related benefits to people (4), but a close daily relationship with dogs or cats facilitate contagion, particularly in children and vulnerable subjects of all ages with impaired immune function (2). Dogs and cats may show clinical signs or be subclinical carriers of the most common zoonotic species, *Microsporum canis*; and *Nannizzia gypsea* and *Trichophyton mentagrophytes* can also be isolated (2, 5–7). Signs and symptoms vary greatly with the host–fungus interaction: canine dermatophytosis is usually characterised by typical round alopecic lesions and brittle hairs with occasionally dry seborrhoea, focal or multi-focal crusted dermatitis, kerion and onychomycosis; in cats, alopecic and inflamed lesions are uncommon, and healthy carriers are often found (2). In humans, dermatophytosis manifestations include ringworm lesions and alopecic areas, traditionally named according to the anatomic locations involved by appending the Latin term of the body site after the word *tinea* (i.e., *tinea capitis*) (1). One of the clinical types of *tinea capitis* is known as kerion, and it is characterised by a painful, inflamed, crusty mass and is often associated with purulent drainage and regional lymphadenopathy (8).

This report is focused on a kerion caused by *N. gypsea* in a 2-year-old boy. *Nannizzia gypsea*, formerly known as *Microsporum gypseum* (3), is mainly described in *tinea corporis* and rarely in *tinea capitis* (or kerion), and it had a lower frequency than other species as a cause of dermatophytosis (9). In worldwide literature, there are only two similar cases regarding infants (10, 11). In those cases, diagnosis was performed by conventional methods only without identification of the causative source of infection. In the present report, a “One-Health” approach (12, 13) allowed this limitation to be overcome, obtaining reliable epidemiological results and preventing disease recurrence as never described before in Italy.

## Case Description

**Figure 1:**
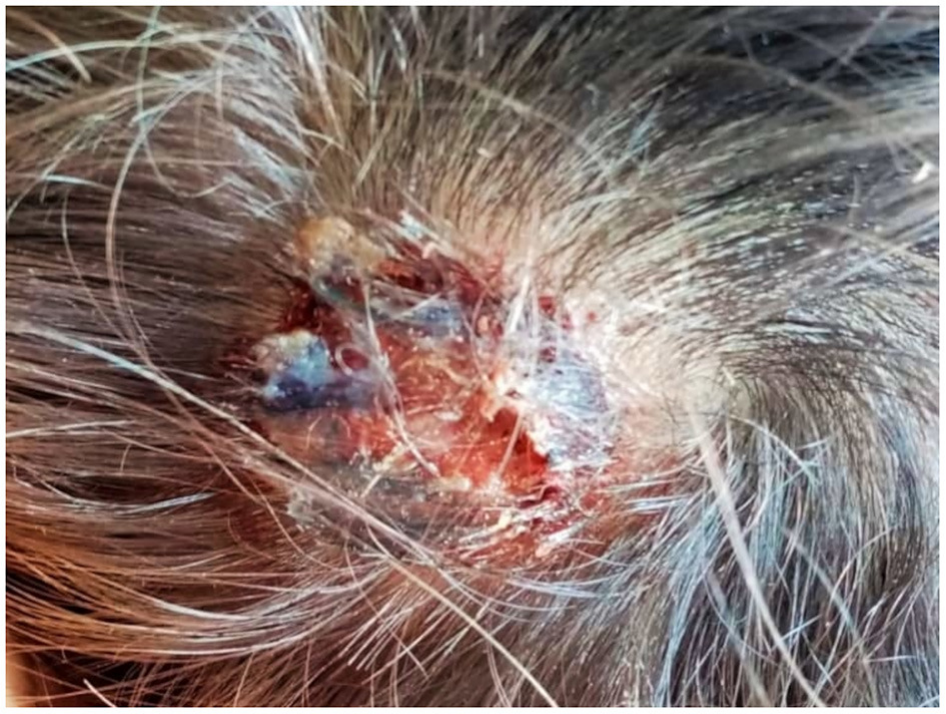
Child's scalp lesion, showing hair loss, pus discharge and crusting.

On August 2020, a 2-year-old boy living in Umbria (Central Italy) showed a round alopecic patch on the temporal-parietal region. Basing on the clinical appearance of the lesion, a dermatologist prescribed daily application of hydrocortisone butyrate (Locoid® Cream), ciclopirox (Milcast® Cream) and amikacin (Dramigel® Gel) creams, without providing a diagnosis supported by mycological culture. As the topical treatment was not effective, oral fluconazole (Diflucan®) was added after about 2 weeks. A few days later, the lesion measured about 4 cm and became swollen, intensely painful and suppurating (Figure 1); and the child presented to the Dermatologic Clinic of Santa Maria Hospital in Terni. Here, considering the clinical aspects and the medical history, the dermatologist recommended mycological exams.

The child was otherwise healthy and immunocompetent. His mother reported that they lived in a country house with child's grandfather and great grandfather. They also kept several kinds of animals, including two dogs, one cat and some hens, which lived in the garden, in the flat and in the henhouse, respectively.

## Diagnostic Assessment

**Figure 2:**
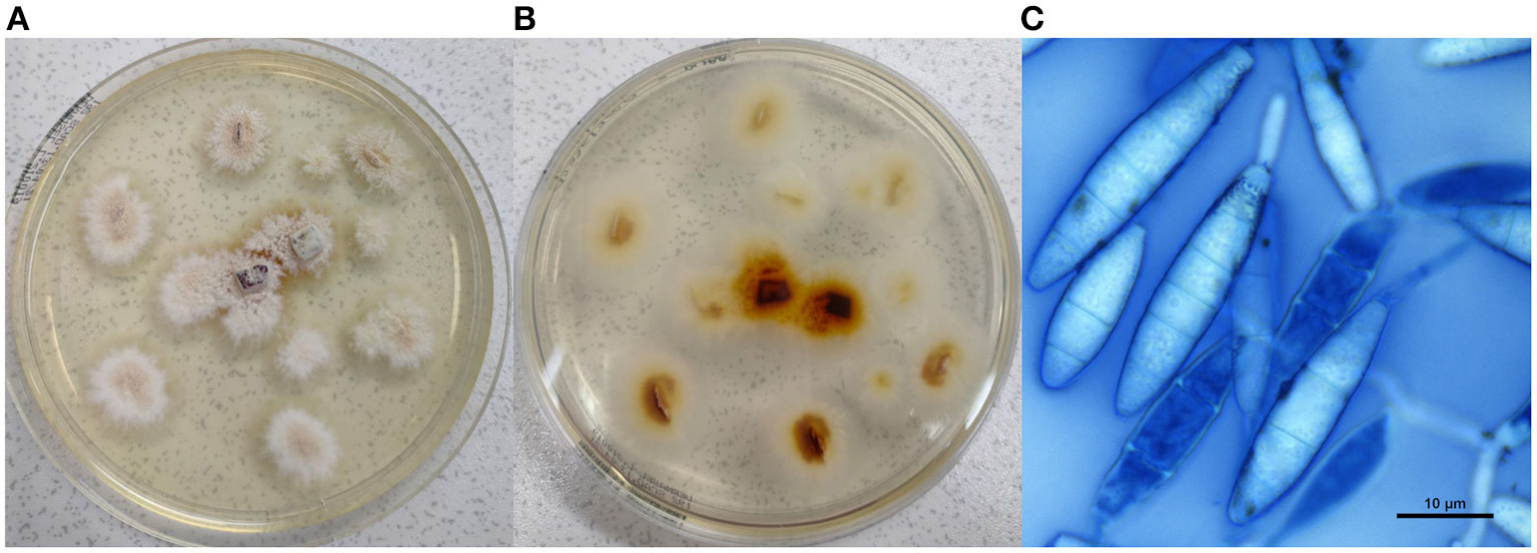
Culture examination. **(A)** Macroscopic appearance of the colonies; **(B)** *reverse* of the macroscopic colonies; **(C)** microscopic view of *Nannizzia gypsea*, showing hyaline, multiseptate and fusiform thin-walled macroconidia (methylene blue stain, × 100, bar 10 μm).

A scalp swab and some hair surrounding the child's lesion were collected for diagnostic purpose and transferred to the Specialist Centre for Fungal Diseases at the Istituto Zooprofilattico Sperimentale dell'Umbria e delle Marche “Togo Rosati.” Samples were inoculated on Dermasel Agar, incubated at 25 ± 1°C and monitored daily. Six days later, a fungal growth consistent with a dermatophyte was observed. The colonies had a macroscopic powdery aspect: they appeared sand-coloured in the central portion and showed a narrow white peripheral border (Figure 2A), while the *reverse* had a brown tint (Figure 2B). Microscopic evaluation was performed through methylene blue staining: abundant hyaline, multiseptate and fusiform thin-walled macroconidia were observed (Figure 2C) as well as hyaline and multiseptate hyphae; just few single-celled and clavate smooth-walled microconidia were noted. PCR and DNA sequencing were performed to identify the fungal species. DNA was extracted using QIAamp DNA mini kit (QIAGEN®, Valencia, CA, USA) following a modified Gram-positive protocol (Appendix D: Protocols for Bacteria, Isolation of genomic DNA from Gram-positive bacteria) and subjected to hemi-nested PCR using DMTF18SF1 (5′-CCAGGGAGGTTGGAAACGACCG-3′) as the common forward primer and DMTF28SR1 (5′-CTACAAATTACAACTCGGACCC-3′) and DMTFITS1R (5′-CCGGAACCAAGAGATCCGTTGTTG-3′) as the reverse primers for the first and second steps, respectively (14, 15). Electrophoresis on 2% agarose gel stained with Midori Green Advance (NIPPON Genetics®, Düren, Germany) showed an ~400-bp amplicon. It was purified by QIAquick PCR Purification Kit (QIAGEN®) and then subjected to Sanger sequencing using BigDye Teminator v3.1 Cycle Sequencing Kit (Thermo Fisher Scientific®, Waltham, MA, USA) and 3,500 Genetic Analyzer (Applied Biosystems®, Foster City, CA, USA). Consensus sequence was created by BioEdit Sequence Alignment Editor software v 7.0.9.0 and then aligned in GenBank database. The strain was identified as *N. gypsea* (GenBank accession number MW691125) with 99% query cover, 0.0 E-value, and 100.00% identity with *N. gypsea* ATCC24102 (Accession No. EF631611).

Additional investigations were carried out by veterinarians in the domestic environment, including the immediate family members, the animals and their habitat and a sample of objects in the flat and in the garden that could be touched by both child and animals (Table 1). Hair samples were collected from family members and animals through Mackenzie technique (16), even if they had no lesions; swabs were used to sample the selected objects. All samples were submitted to the same analysis process as described above. Fungal growth was obtained from dog hair, doghouse and indoor carpet: the strains were identified as *N. gypsea* (GenBank accession numbers MZ437979, MZ437947, MZ437978, and MZ435341) with 99% query cover, 0.0 E-value, and 100.00% identity with *N. gypsea* ATCC24102 (Accession No. EF631611).

**Table 1:** Details of veterinarians' sampling and laboratory findings.

**Hair coat samples**	**Indoor samples**	**Outdoor samples**	**Environment**	**Mycological exam**	**DNA sequencing**
Dog 1				+	*Nannizzia gypsea* (MZ437979)
Dog 2				+	*Nannizzia gypsea* (MZ437947)
Cat				-	
	Cat bed		1st floor	-	
	Carpet 1		1st floor	-	
	Carpet 2		1st floor	-	
	Sofa		1st floor	-	
	Carpet		Ground floor	+	*Nannizzia gypsea* (MZ435341)
	Sofa		Ground floor	-	
		Dog bowl 1	Garden	-	
		Dog bowl 2	Garden	-	
		Doghouse	Garden	+	*Nannizzia gypsea* (MZ437978)
		Tennis ball	Garden	-	
		Barrow	Garden	-	
		Manger	Henhouse	-	
		Firewood	Woodshed	-	
		Mouse trap	Tool shed	-	
		Sand	Tool shed	-	

The child was treated with both systemic and topical therapy, without adverse or unanticipated effects. Oral micronized griseofulvin (Fulcin®) at a dosage of 25/mg/kg/day (125 mg TID) was recommended for 8 weeks. Dressings with a cream containing isoconazole and diflucortolone (Travocort® 0.1 + 1% Cream) were used in the first week in order to quickly relieve inflammation and related symptoms, followed by daily dressings with terbinafine cream (Lamisil® 1% Cream) until complete clinical and mycological healing of the lesion occurred.

**Figure 3:**
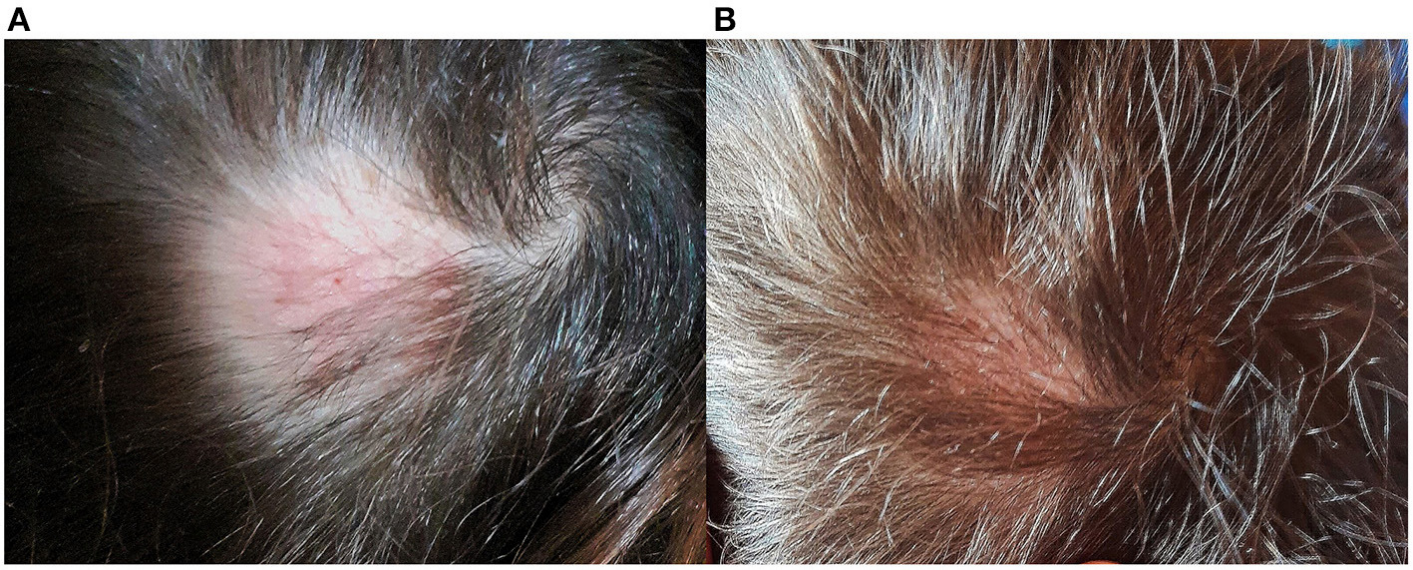
Clinical course. **(A)** Three months and **(B)** 4 months after initiation of treatment.

Dogs were subjected to chlorhexidine and miconazole baths (Malaseb® 20 + 20 g/L of shampoo, 1 bath/week) for 5 weeks. From the third month after initiation of dual treatment to the child and dogs, culture examination for the dermatophyte became negative, and the child's hair gradually grew back (Figures 3A,B).

## Discussion

In the current case, a “One-Health” approach based on dermatologists' and veterinarians' collaboration has been useful to identify both the causative agent and the most probable causative source of the fungal infection. Searching for the causative source of contamination by mycological examination of pets has been done for many years when cases of *tinea* caused by anthropophilic and zoophilic dermatophytes are diagnosed (17). The authors commonly use the One-Health approach when human dermatophytosis occurs, whatever the natural reservoir of the dermatophyte is. To standardise this approach, they always carry out the same procedure when humans cohabit with animals, performing both pet and environment sampling. This multidisciplinary approach allows to (i) successfully heal the fungal infection, (ii) implement prophylactic measures, and (iii) prevent disease recurrence once remission has been achieved.

The causative agent of the kerion was identified through conventional and molecular-based techniques, which ensured reliable identification of *N. gypsea*, overcoming the limitation of conventional methods in the fungal discrimination compared with the most recent introduced taxonomy (3): macroscopic and microscopic features of the isolated colonies may currently not be typical of a single strain giving imprecise results (18). In literature, *N. gypsea* has already been described as causative agent of *tinea capitis* and kerion, even if it has a low frequency as compared with anthropophilic and zoophilic dermatophytes (8, 9, 19, 20). As described by other authors (19), the 0–9 age range of presenting children show the highest percentage of infections due to *N. gypsea*, with a slight male preponderance, attributable to the common practise of boys playing outdoor more often than girls.

Veterinarians' investigations in the domestic environment identified healthy carrier dogs as one of the possible sources of infection. Dogs as healthy carriers of *N. gypsea* have been already described (21, 22), with higher percentages compared with dogs showing lesions (23). Dogs living outdoor are easily predisposed to geophilic organism contamination because of their “burrowing and denning” habits (21, 23). According to Rabinowitz et al., dogs may be less infectious than cats (24). Romano et al. isolated *N. gypsea* in both sick and healthy carrier cats responsible for human dermatophytosis (7). In the current case, the cat tested was surprisingly negative for *N. gypsea*, probably because it remained upstairs and had no contact with contaminated soil or carrier dogs. Veterinarians' investigations also discovered fungal contamination in the doghouse and within an indoor carpet, placed downstairs in an area accessible to dogs: these items were thrown away to prevent recurrences or new infections.

The authors supposed that the child could have injured himself on the scalp by playing outdoor, facilitating fungal penetration. Considering that *N. gypsea* is a geophilic dermatophyte and the outcomes of the veterinarians' investigations, the child's infection could have occurred by contact with soil and/or infected objects but otherwise *via* contaminated dog hair. However, it was reasonable to think that the child could be more exposed to fungal spores carried by the dogs given the close contact and the time spent playing with them.

The child's lesion successfully healed with systemic and topical therapy. Previous topical treatments with hydrocortisone butyrate, ciclopirox and amikacin creams were not effective, like oral fluconazole, which was probably low rated. In addition, the lesion worsened after a few days of treatment, becoming intensely painful and suppurating. Griseofulvin has traditionally been the most widely prescribed and commonly used antifungal treatment for *tinea capitis* in clinical practise (25, 26). Thus, the dermatologist based systemic therapy on micronized griseofulvin in association with terbinafine and prescribed additional topical treatments with isoconazole-diflucortolone as suggested by Schaller et al. (27). The child's lesion began to regress after about 8 weeks, and his hair gradually grew back. The clinical findings confirmed that scarring is less severe and less frequent after resolution as compared with more historic cases, probably because of the more rapid resolution of the disease with oral antimycotic drugs (28).

Since the dogs did not show skin lesions, the veterinarians opted for a topical treatment. Some authors suggest a combination of topical treatments with systemic griseofulvin for a more effective result (23); in this case, chlorhexidine and miconazole baths proved adequate.

It would be desirable for human dermatologists to follow this multidisciplinary procedure in order to improve mycological diagnosis, prognosis and epidemiology and to also avoid mistakes or delays in diagnosis and possible use of unsuitable therapies. This multidisciplinary approach may aid prevention of permanent and unaesthetic lesions. Veterinarian contributions should be increasingly recommended in such cases to investigate and identify potential causative sources of infection and to treat them to prevent disease recurrence, the success of which was highlighted in our approach.

## Data Availability Statement

The original contributions presented in the study are included in the article/supplementary files, further inquiries can be directed to the corresponding author.

## Ethics Statement

Ethical review and approval was not required for the study on human participants in accordance with the local legislation and institutional requirements. The animals were not part of an animal testing and were subjected to noninvasive sampling procedures. Written informed consent was obtained from the owners for the participation of their animals in this study and for the publication of any potentially identifiable images or data included in this article.

## Author Contributions

DC drafted the report. MP and SC managed human and veterinarian therapeutic assessment, respectively. DC, SB, SS, and YN collected data. MP, SC, and FA reviewed and edited the manuscript. All authors contributed to data interpretation and approved the manuscript.

## Conflict of Interest

The authors declare that the research was conducted in the absence of any commercial or financial relationships that could be construed as a potential conflict of interest.

## Publisher's Note

All claims expressed in this article are solely those of the authors and do not necessarily represent those of their affiliated organizations, or those of the publisher, the editors and the reviewers. Any product that may be evaluated in this article, or claim that may be made by its manufacturer, is not guaranteed or endorsed by the publisher.
